# Caffeine, D-glucuronolactone and Taurine Content in Energy Drinks: Exposure and Risk Assessment

**DOI:** 10.3390/nu14235103

**Published:** 2022-12-01

**Authors:** Carmen Rubio, Montaña Cámara, Rosa María Giner, María José González-Muñoz, Esther López-García, Francisco J. Morales, M. Victoria Moreno-Arribas, María P. Portillo, Elena Bethencourt

**Affiliations:** 1 Toxicology Department, Pharmacy and Health Sciences Faculties, Universidad de La Laguna (ULL), 38071 San Cristóbal de La Laguna, Spain; 2 Nutrition and Food Science Department, Pharmacy Faculty, Complutense University of Madrid (UCM), Plaza Ramón y Cajal, s/n, 28040 Madrid, Spain; 3 Department of Pharmacology, Faculty of Pharmacy, University of Valencia, 46100 Burjassot, Spain; 4 Department of Biomedical Sciences, Toxicology Unit, Faculty of Pharmacy, University of Alcalá, Alcalá de Henares, 28871 Madrid, Spain; 5 Department of Preventive Medicine and Public Health, School of Medicine, Universidad Autónoma de Madrid, Spain/IdiPAZ, CIBER of Epidemiology and Public Health (CIBERESP), 28029 Madrid, Spain; 6 Institute of Food Science, Technology and Nutrition (ICTAN-CSIC), 28040 Madrid, Spain; 7 Institute of Food Science Research (CIAL), CSIC-UAM, 28049 Madrid, Spain; 8 Nutrition and Obesity Group, Department of Pharmacy and Food Sciences, University of the Basque Country (UPV/EHU), 01006 Vitoria-Gasteiz, Spain; 9 CIBERobn Physiopathology of Obesity and Nutrition, Institute of Health Carlos III, 28029 Madrid, Spain; 10 BIOARABA Institute of Health, 01009 Vitoria-Gasteiz, Spain; 11 Elena Bethencourt Barbuzano, Toxicology Department, Pharmacy and Health Sciences Faculties, Universidad de La Laguna (ULL), 38071 San Cristóbal de La Laguna, Spain

**Keywords:** energy drinks, caffeine, taurine, D-glucuronolactone, exposure assessment, risk characterization, risk management

## Abstract

The consumption of energy drinks (EDs) is increasing globally while the evidence and concern about the potential health risks are also growing. Caffeine (generally 32 mg/100 mL) together with a wide variety of other active components such as taurine (usually 4000 mg/L) and D-glucuronolactone (generally 2400 mg/L) are the main ingredients of EDs. This study aims to assess the exposures to caffeine, taurine and D-glucuronolactone from EDs in various consumption scenarios and consumer profiles and to characterize the risks by evaluating caffeine and taurine intakes with their reference values and by calculating the margin of safety (MOS) for D-glucuronolactone. While the exposure assessment results showed that caffeine intakes from EDs ranged from 80 to 160 mg (1.14–4 mg/kg b.w.) for the considered scenarios, the risk characterization estimated some risks that could be managed with consumption recommendations such as limiting EDs in 40, 60 and 80 kg b.w. consumers to 175, 262.5 and 350 mL, respectively, to prevent sleep disturbances and to 375, 562.5 and 750 mL to prevent general caffeine adverse health risks, respectively. Dietary exposure to D-glucuronolactone from EDs ranged from 600 to 1200 mg (7.5–30 mg/kg b.w.). As D-glucuronolactone MOS ≥ 100 is only observed when EDs consumption is limited to 250 mL, for individuals weighing above 60 kg, some risks were observed in some of the studied scenarios. A taurine exposure from EDs varied from 1000 to 2000 mg (12.5–50 mg/kg b.w.) and consumptions over 500 mL were estimated to generate intakes above the reference value. In conclusion, the management of these risks requires a European legal framework for EDs with maximum limits for the active components, volume size limitations and labeling improvements along with the development of education and awareness programs and risk communication actions in collaboration with the industry and society.

## 1. Introduction

The energy drinks (EDs) market is estimated to be 1% of the non-alcoholic drinks market and this may be attributed to the well-thought-out design process in the food industry. The consumption of EDs has considerably increased worldwide [1,2,3,4,5,6,7,8,9,10,11], especially among male adolescents and young adults. The European FoodEx2 classification classifies EDs in the non-alcoholic functional drinks category [12].

EDs consumption was estimated to be at 30% in European adults (18–65 years; 16% corresponding to chronic consumers) and at 68% in European adolescents (10–18 years; 10% classified as chronic consumers). Almost 12% of all adult Europeans (13.3% of “young adults”) described themselves as regular consumers, drinking EDs 4–5 times a week or more and consuming a mean average volume of 4–5 L/month [13]. The Spanish Survey on Drug Use in Secondary Schools (ESTUDES) estimates the prevalence of EDs use among students at 50.7% and 39% for young males and females, respectively [14].

Recent studies report that, during the COVID-19 pandemic, not only has the frequency of the consumption of EDs increased but also the amount ingested, which was probably due in part to the need to cope with stress, boredom and the desire to improve attention when using screens and playing video games [15,16].

There is a global growing concern about the potential risks and the existing low-risk perception associated with these drinks [2,9,10,17,18,19]. In general, the evidence correlates EDs with a significant increase in the odds of insomnia (and jitteriness/activeness) [11], anxiety, depression, impulsivity and poor academic performance, among others. While frequent Eds consumption generates stimulation (nervous and cardiovascular), hypertension, bone density loss, osteoporosis, low psychological, physical, educational and overall well-being, among other consequences [2,11,20,21], acute Eds consumption not only generates a caffeine overdose but has also been identified as an indicator of the use/abuse of other psychoactive substances (tobacco, sedatives, cannabis, cocaine and ecstasy) and risky behaviors [1,14,17,22,23,24]. The combined consumption of EDs with alcoholic drinks is known to generate, among other effects, a decreased sense of drunkenness [17], and this has been a growing cause of concern since more than half of young European consumers said they occasionally consumed EDs mixed with alcohol [13].

Despite the variety of ingredients, most EDs share the same composition of caffeine, taurine and D-glucuronolactone in varying proportions, along with other minor components such as B vitamins and L-carnitine that increase their attractiveness, especially among young consumers [25,26]. While the European Food Safety Authority (EFSA) found that forty-nine of the fifty-three EDs distributed in Europe contained taurine in their formula [13], the French Agence Nationale de Sécurité Sanitaire de l’alimentation, de l’environnement et du travail (ANSES) reported that only 103 out of 126 of the EDs marketed in France showed a complete list of ingredients on the packaging and, of these, only 52% contained taurine [27]. Additionally, 33% of the EDs on the French market contained D-glucuronolactone and 59% of the EDs did not state the amount on the labeling [27].

The caffeine content in EDs usually ranges between 15 and 55 mg/100 mL [28], although the most common concentration is 32 mg/100 mL. A standard EDs formula usually contains 2400 mg/L of D-glucuronolactone and 4000 mg/L of taurine [26,28,29]. Nevertheless, the mean average taurine content in EDs has progressively increased since the first commercialized formulations [30]. While ANSES reported a mean taurine content (mg/L) of 3800 [27], EFSA reported 3412 [29] and Health Canada reported a mean average of 4000 (range: 40–8000 mg/L) [31]. The mean D-glucuronolactone content (mg/L) was 1700 (range: 240–2400) but according to Health Canada, the D-glucuronolactone content may be up to 4800 [31].

Caffeine (1,3,7-trimethylxanthine) generates multisystemic effects not only in the central nervous system (CNS) but also in the respiratory, renal, endocrine, urinary, musculoskeletal and cardiovascular systems [32,33,34]. Caffeine, in the CNS, behaves as an antagonist of adenosine A1, A2A and A2B receptors, producing a mild excitatory effect [35,36,37]. Caffeine increases natriuresis and diuresis by an interaction with the A1 receptor [28] and inhibits phosphodiesterase, causing a smooth muscle relaxation [35,38,39]. A positive chronotropic and inotropic effect has been described at a cardiovascular level, as well as arrhythmias, tachycardias and an increase in blood pressure and heart rate [26,32,40]. In addition, caffeine is known for its potential to cause moderate physical dependence and tolerance. The EFSA associates intakes (mg/caffeine/kg b.w./day) of three with general effects (cardiovascular and hematological, neurological and psycho-behavioral) and of 1.4 with sleep disturbances (sleep onset latency and shorter duration) [35]. However, the Norwegian Scientific Committee for Food Safety [28] recently concluded that the benchmark of 3 mg caffeine/kg b.w./day may not necessarily protect against certain cardiac ailments. In any case, according to EFSA, general consumers (70 kg b.w.) should keep their caffeine ingestion under 400 mg/day [35].

The provision of information to consumers in Europe [41] includes labeling requirements for beverages with a high caffeine content (>150 mg/L), such as displaying *“High caffeine content. Not recommended for children or pregnant or breastfeeding women”* along with the caffeine amount in mg/100 mL. It is worth mentioning that some European brands [42] include *“Consume Moderately”* or similar wording on their labels and others follow a voluntary code where labels are committed to not promoting the combined use with alcoholic beverages [42,43].

One of the most recent risk communication actions on EDs has been promoted and executed by the Spanish Agency of Food Safety and Nutrition (AESAN) in 2022 [44]. As a risk management action, the AESAN, following a 2021 risk assessment scientific report [26], published a document in 2022 with recommendations on the consumption of EDs [44]. These recommendations remind athletes that EDs are not designed for rehydration and should not replace hydration and the recovery of metabolites by the conventional means such as water or, where appropriate, through isotonic drinks. They also say that the regular consumption of caffeine (100 mg/day) may cause a moderate physical dependence and tolerance, that an excessive caffeine consumption may have negative physiological effects and that EDs should not be combined with alcoholic beverages [44]. The AESAN has also recommended avoiding their consumption in case of children, adolescents, pregnant and breastfeeding women, people with hypertension, cardiovascular problems or with sleep disorders [44]. Finally, the AESAN risk communication campaign points out that EDs with sugars may contribute to exceeding the daily intake recommendations of simple sugars (the WHO recommendation: 50 g/day) since 250 mL of EDs may contain between 27.5 and 30 g of sugars and 500 mL of EDs between 55 and 60 g of sugars.

Although the dietary exposure to D-glucuronolactone is generally estimated to be low (1–2 mg/day) [29,36], the detection of unspecified renal lesions (renal papilla inflammation) in rats during the hazard identification of D-glucuronolactone raised concerns about the safety of including this ingredient in EDs [45]. The lowest no-observed-adverse-effect level (NOAEL) for these nephrotoxic effects was initially set at 300 mg/kg b.w./day, but based on the subsequent histopathological findings regarding renal inflammation, the NOAEL was finally set at 1000 mg/kg b.w./day [29].

Taurine (2-aminoethanesulfonic acid) is found in high concentrations in cardiac muscle and the CNS, although its levels decrease significantly with age. Taurine, unlike caffeine, behaves as an inhibitory neuromodulator. Its antioxidant and anti-inflammatory properties suggest its participation in several biological processes (the stabilization of the plasma membrane and bile salts, osmoregulation, calcium metabolism, skeletal muscle functionality and correct neuronal activity, among others) [46,47,48], but few studies have related dietary exposure with cardiovascular and neurological effects [49,50,51,52]. There are several dietary sources of taurine [36,46] that contribute to the estimated daily taurine intake (10–400 mg/day) [30] but, depending on the type of diet, the dietary intake may be lower (20–200 mg/day) [53]. In the case of omnivorous diets, the daily intake is estimated to be at 58 mg of taurine/day [29]. Based on the taurine hazard characterization, the EFSA established a daily reference intake of 1400 mg taurine/day for a 70 kg b.w. individual [29].

A taurine supplementation has been associated with a potential protective activity in aging brains and direct beneficial effects during nervous system toxicity episodes [54,55,56,57], but the EFSA states that a taurine dietary intake does not increase the taurine levels in the brain, ruling out the possibility of a stimulant effect on the CNS [29]. Therefore, a taurine supplementation may not be necessary in healthy consumers. Although Health Canada reported that acute taurine oral toxicity is low [58], and excess taurine may generate cognitive and behavioral effects in young adults [59].

Because an exposure to D-glucuronolactone and taurine has raised safety concerns, especially in high and chronic consumption scenarios [60], individual initiatives have been launched in different countries, such as Germany and Denmark [61], promoting the standardization of EDs with maximum caffeine levels at 32 mg/100 mL; taurine levels at 4000 mg/L; and glucuronolactone contents at 2400 mg/L [62].

Given this background, the objectives of the present assessment were to estimate the dietary exposures to caffeine, D-glucuronolactone and taurine derived from EDs in various consumption scenarios and for various consumer profiles, to characterize the potential health risks and to suggest some recommendations for risk management and communication.

## 2. Materials and Methods

The dietary exposure assessment (estimated daily intake, EDI) (1) of the three components under study was conducted by studying different volumes of ED containers marketed around the world (250, 333 and 500 mL) and the standard levels of caffeine (32 mg/100 mL), taurine (4000 mg/L) and D-glucuronolactone (2400 mg/L) in the above-mentioned commercial presentations. In addition, three consumption scenarios (250, 333 and 500 mL/day) and three consumer profiles based on body weight (40, 60 and 80 kg) were evaluated.

EDI = C of caffeine/D-gluconolactone/taurine (mg/L) · V of ED (L)(1)

C: concentration; V: volume.

A 1000 mL (1 L) consumption scenario was not considered as it was considered to be unusual among general consumers even though, according to Zucconi et al., 11% of all adult and 12% of all adolescent consumers of EDs were excessive consumers ingesting at least 1 L in a single ingestion [13].

The risk characterization for caffeine and taurine was performed by evaluating the caffeine and taurine estimated daily intakes (EDIs) with the established reference intakes for caffeine (intake > 1.4 mg/kg b.w./day leads to sleep disturbances; intake > 3 mg/kg b.w./day causes general adverse effects (cardiovascular and hematological, neurological and psycho-behavioral effects) [35] and taurine (1400 mg/day) [29].

In regard to D-glucuronolactone, the risk characterization was performed by calculating the margin of safety (MOS) using the no observed adverse effect level (NOAEL) and the estimated daily intake (EDI) (2) [63].

(2) $$\mathrm{MOS}=\frac{\mathrm{NOAEL}\;(\mathrm{mg}/\mathrm{kg}\;\mathrm{of}\;\mathrm{body}\;\mathrm{weight}/\mathrm{day})}{\mathrm{EDI}\;(\mathrm{mg}/\mathrm{kg}\;\mathrm{of}\;\mathrm{body}\;\mathrm{weight}/\mathrm{day})}$$

An acceptable value of the MOS for a NOAEL-based assessment extrapolated from an animal study is ≥100 (factor 10 for an extrapolation from animals to humans and a factor 10 for interindividual variation in humans). The D-gluconolactone NOAEL is set by EFSA at 1000 mg/kg b.w./day [29].

## 3. Results and Discussion

### 3.1. Caffeine: Exposure Assessment and Risk Characterization from EDs

Energy drinks are generally marketed worldwide in three standard volumes (250, 333 and 500 mL) and the most common caffeine content is 32 mg/100 mL. Considering these data, the estimated daily caffeine intakes for the three consumer profiles (40, 60 and 80 kg body weight) under evaluation are shown in Table 1.

The caffeine estimated daily intakes (EDIs) from EDs ranged from 80 mg when 250 mL are consumed to 160 mg when 500 mL are ingested. These results are higher than those previously reported by Zucconi et al. and the Norwegian Ungkost 3 study [13,28]. According to Zucconi et al., the daily caffeine exposure was estimated at 22.4 mg (0.32 mg/kg b.w.) for adult European consumers and at 48.3 mg (0.7 mg/kg b.w.) for high chronic consumers. Likewise, a daily caffeine exposure was estimated at 23.5 mg (0.38 mg/kg b.w.) in European adolescents (10–18 years), increasing to 75.08 mg (1.18 mg/kg b.w.) in chronic high adolescent consumers [13]. In 2015, the mean average daily caffeine intakes in adults were estimated again, after observing a wide variability among EU Member States, obtaining a caffeine intake of 37–319 mg [35]. More recently the Norwegian Ungkost 3 study estimated the dietary caffeine exposure from Eds at 36.8 mg caffeine/day [28].

For a 40 kg person, daily caffeine intakes are estimated to be 2, 2.7 and 4 mg/kg b.w. when consuming 250, 333 and 500 mL, respectively. In the risk characterization, considering the limit values established by the EFSA for sleep disorders (1.4 mg/kg b.w./day), the authors conclude that any consumption equal to or higher than 250 mL will expose the consumer to the risk of sleep disorders. Similarly, intakes of 500 mL will expose the 40 kg b.w. consumer to levels over the 3 mg caffeine/kg b.w./day that the EFSA has correlated not only with sleeping disturbances but also with general adverse health effects [35].

For a 60 kg individual, a daily caffeine intake is estimated to reach 1.3, 1.8 and 2.6 mg/kg b.w. after consuming 250, 333 and 500 mL of EDs, respectively. Based on the EFSA health-based limit of 1.4 mg/kg b.w./day for sleep disorders, the risk characterization suggests limiting the consumption of EDs to 250 mL to avoid the risk of sleep disorders. However, the risk characterization concludes that any EDs consumption below 500 mL will keep one’s caffeine intake below 3 mg caffeine/kg b.w./day, thereby avoiding the overall adverse health effects [35].

In the highest body weight scenario considered (80 kg), the daily estimated caffeine intakes are 1.14, 1.3 and 2 mg/kg b.w. after consuming 250, 333 and 500 mL of EDs, respectively. Based on the health-based limit of 1.4 mg/kg b.w./day for sleep disturbances, the risk characterization suggests limiting the consumption of EDs to 333 mL to avoid the risk of sleep disturbances. No overall adverse health effects are expected for any of the three consumption scenarios.

ED consumers undoubtedly have a total caffeine intake that exceeds that observed for non-EDs consumers [7]. As previously established by Ruiz and Scherr, Zucconi et al. and Ungkost 3 (Norwegian Ungkost 3 Study), the results here show a trend of an increased dietary caffeine exposure due to the increasing consumption of EDs [13,23,28]. Nevertheless, as reviewed by Verster and Koenig, caffeine intake is generally below the recommended levels [64]. However, the authors suggest enhancing the use of these consumption recommendations based on the upper intake limits proposed by the EFSA in the education, communication and management of the risks associated with EDs. All stakeholders should also be encouraged to contribute by applying education and communication strategies to minimize the risks associated with caffeine and to promote the moderate consumption of EDs considering the diversity of the consumers.

Table 2 suggests different ED consumption limits according to the different body weight profiles considered in this assessment. To prevent sleep disorders, the consumption of EDs should be limited to 175, 262.5 and 350 mL in consumers of a 40, 60 and 80 kg body weight, respectively. To prevent general adverse health effects, Eds formulated with 32 mg caffeine/100 mL should be limited to 375, 562.5 and 750 mL in consumers of a 40, 60 and 80 kg body weight, respectively.

### 3.2. D-glucuronolactone: Exposure Assessment and Risk Characterization from EDs

Table 3 shows the estimated dietary intakes (EDI) of D-glucuronolactone from EDs formulated with 2400 mg/L. For the risk characterization of the dietary exposure to D-glucuronolactone from EDs, the margins of safety (MOS) were estimated considering the NOAEL of 1000 mg/kg b.w./day [29]. As mentioned above, an acceptable value of the MOS for an NOAEL-based assessment extrapolated from an animal study is ≥100.

A dietary exposure to D-glucuronolactone from EDs ranges from 600 to 1200 mg depending on the volume of EDs with 2400 mg D-glucuronolactone/l consumed (250–500 mL). Considering the different body weight profiles proposed (40, 60 and 80 kg), a dietary exposure to D-glucuronolactone from EDs is estimated to vary from 7.5 to 30 mg/kg b.w.

Although the results here are higher than those previously reported for European populations by Zucconi et al.: adolescent (100.14 mg/day = 1.65 mg/kg b.w./day), adult (125.95 mg/day = 1.78 mg/kg b.w./day), chronic high adolescent EDs consumers (311.6 mg/day = 4.9 mg/kg b.w./day) and chronic high EDs consumers (268.84 mg/day = 3.9 mg/kg b.w./day). The results here are similar to those estimated for acute Spanish consumers by Zucconi et al.: adult 906.32 mg/day (12.87 mg/kg b.w./day) and 143 mg/day (2.02 mg/kg b.w./day) in acute and chronic use of EDs, respectively; adolescents 551.49 mg/day (9.56 mg/kg b.w./day); and 74.50 mg/day (1.27 mg/kg b.w./day) in an acute and chronic consumption, respectively [13]. It is not possible to compare the results of the present study with those published by the Norwegian Food Safety Agency [65] as this Agency estimated the mean average intake from Eds considering a 240 mg/L D-glucuronolactone content.

The risk characterization performed in the present study by estimating the margin of safety (MOS) suggests that the consumption of a high volume of EDs (up to 500 mL) reduces the MOS. Individuals weighing 60 and 80 kg would only present an MOS ≥100 when their consumption of Eds with 2400 mg of D-glucuronolactone/l is limited to 250 mL, although in the latter case this is also observed when their consumption is 333 mL in 80 kg individuals. These results do not support the EFSA statement based on the NOAEL established for the toxicological effects of D-glucuronolactone (1000 mg/kg b.w./day) which reported that dietary exposures at the levels present in EDs are not a health concern for a person of a 60 kg body weight, even when the chronic consumption of EDs is high (350 mL/day) [29]. Finally, an MOS <100 was estimated in all three consumption scenarios (250, 333 and 500 mL) for those individuals with low body weights (around 40 kg) so the health risks from the exposure to the D-glucuronolactone contents in EDs might be expected.

### 3.3. Taurine: Exposure Assessment and Risk Characterization from EDs

Considering a mean taurine content in EDs of 4000 mg/L, Table 4 shows the estimated taurine exposure under three EDs consumption scenarios (250, 333 and 500 mL) and three body weights (40, 60 and 80 kg b.w.). Along with a taurine exposure, the risk is characterized considering the reference EFSA intake for taurine set at 1400 mg/day [29,66].

A taurine exposure from EDs varies from 1000 to 2000 mg depending on the volume of the EDs consumed. The acute taurine exposure estimated in the assessment here (2000 mg/day) is similar to the acute taurine exposure previously assessed for the European population (1851 mg/day and 1809 mg/day for adults and adolescents, respectively) [13]. However, the intake estimates are higher than those previously reported for chronic consumers [13] not only for adult Europeans (271.9 mg/day (3.82 mg/kg b.w./day)–585.8 mg/day (8.49 mg/kg b.w./day)) but also for adolescents (283.9 mg taurine/day (4.6 mg/kg b.w./day)–924.3 mg taurine/day (14.5 mg/kg b.w./day)). The results are also higher than the mean daily taurine intakes estimated by the ANSES, 181 mg/day (3.02 mg/kg b.w./day; b.w. = 60 kg) for all consumers, 429 mg/day (7.5 mg/kg b.w./day; b.w. = 60 kg) for regular users; and 714 mg/day (53.57 mg/kg b.w./day; b.w. = 60 kg) for chronic users (P90)). The data here are at least five times higher than those reported by the EFSA for adults for Spain in 2013, where a daily taurine exposure from the consumption of EDs was estimated at 290 mg and 149 mg in adults and adolescents, respectively [13]. A possible explanation for this growing dietary exposure from EDs is, as mentioned above, the progressively increasing taurine content in EDs since the first commercialized formulations [30].

According to the present study, a daily EDs ingestion of 500 mL exposes the consumer to a daily dietary intake of 2000 mg of taurine, which exceeds the EFSA daily recommendation of 1400 mg taurine [29]. Considering the different body weight profiles and Eds as the only dietary source of taurine, the assessment here estimates that the taurine exposure from EDs varies between 12.5 mg/kg b.w./day for 80 kg and 50 mg/kg b.w./day for 40 kg.

Considering EDs as the only dietary source of taurine, the following risk characterization was assessed:

In 40 kg b.w. individuals, while the 250 and 333 mL consumption scenarios keep the taurine intake from EDs below the reference value established by the EFSA (35 mg/kg b.w/day), consuming 500 mL will expose the individual to intakes above the reference intake, posing a health risk that may require management and communication measures, such as those proposed above for caffeine.

In 60 kg b.w. individuals, the estimated dietary intake (EDI) ranges from 16.7 to 33.3 mg/kg b.w. Therefore, the 250 and 333 mL consumption scenarios will ensure that the consumer keeps the taurine intake below the 23.3 mg/kg b.w./day considered as the reference value.

In 80 kg b.w. consumers, the estimated dietary intake (EDI) when consuming 250 and 333 mL of EDs would be below the reference value of 17.5 mg/kg b.w./day and no risks are to be expected. However, as before, a 500 mL consumption will expose the consumer to exceeding the reference value and suffer the associated health risks.

Health Canada’s health risk assessment concluded that two ED units (250 mL) could be safely consumed each day without negative health effects [58] because the acute oral toxicity of taurine is considered to be relatively low.

The results here are similar to those previously reported by the VKM taurine risk assessment from Eds and food supplements [65]. According to this agency, the dietary intakes in the chronic Eds intake model were all below the reference value and it was unlikely that a chronic taurine intake could cause adverse health effects. However, the abovementioned agency considered that a chronic high taurine intake from EDs could lead to health risks in young children (3 to <10 years) but not to children (10 to 14 years), adolescents (14 to <18 years) or adults [65].

Finally, it is worth mentioning that the uncertainty of the possible effects of a joint taurine and caffeine intake remains unclear, and this may influence a risk assessment as there is a lack of knowledge about the risks of a long-term chronic exposure.

### 3.4. Risk Management

The management of the risks derived from the dietary exposure to these three EDs active components would be strengthened if there was a legal framework for EDs at a European level with the setting of maximum limits for the active components and their possible combinations. The consumption of EDs and the results on health require a more detailed analysis and follow-up as consumption patterns and risk minimization depend on multiple factors, among which sociodemographic factors stand out [4,19]. Different authors and agencies highlight the need to regulate EDs, to limit and moderate their consumption in children and adolescents, to promote the communication of recommendations and risks associated with their consumption, among others [3,6,17,26,65].

The following recommendations stand out among the different strategies proposed to minimize the health risks associated with EDs: limiting and regulating direct marketing [6,17], raising awareness campaigns such as the one promoted by the AESAN in 2022 [44,67], educational programs on the risks of combining alcohol and energy drinks [1,67,68], having awareness and communication campaigns adapted to different genders and ages [69] and the promotion of the follow-up/monitoring of consumption trends [26,67].

Furthermore, in terms of risk management, the authors suggest following the quadruple helix model to enhance an active collaboration between risk managers and regulators with the industry and the community in order to optimize labeling, portion sizes and risk communication, among others. The volume of ED containers varies, reaching up to 500 mL in some cases, but Energy Drinks Europe (EDE) has committed, in its Code of Practice, to the production and marketing of containers with a net content of 250 mL as the main selling proposition [42]. Moving forward with this initiative and limiting/regulating the volume of marketed ED containers to a maximum of 333 mL would be an effective management action to minimize the risks associated with high intakes of EDs.

## 4. Conclusions

The growing concern for assessing the health risks associated with the consumption of EDs and the dietary exposure to their active components has led to the commitment of academia, government, industry and society to increase awareness, knowledge and monitoring. It is undoubtedly necessary to advance in the establishment of a legal framework for EDs in Europe that includes the setting of maximum contents of active ingredients, to monitor the dietary exposures to all the active components and not exclusively caffeine and to improve the information to consumers in collaboration with the industry and society at large.

Regarding labeling, there is much room for improvement, such as indicating the content of each of the ingredients, especially those that may pose a health risk, such as D-glucuronolactone and taurine. Smaller volume packaging should be encouraged because limiting this would contribute to moderating the exposure to the different active components. Furthermore, as stated in the report of the Scientific Committee of the AESAN [26], compliance with the industry’s commitment to marketing packages containing no more than 250 mL is recommended to minimize exposure to the different active ingredients, some of which are psychoactive, and as well as studying the possibility of stopping the marketing of 500 mL packages.

Consumer recommendations on EDs should be included in risk communication and educational campaigns to increase public awareness and risk perception. EDs advertising and marketing should also be regulated.

## Figures and Tables

**Table 1:** Caffeine: dietary exposure assessment and risk characterization when consuming 250, 333 and 500 mL of 32 mg caffeine/100 mL EDs.

	**Volume of ED ingested (mL)**		
	250 mL	333 mL	500 mL
	**Total caffeine intake (mg)**		
	80 mg	107 mg	160 mg
	**Caffeine intake per kg of body weight (mg/kg b.w.)**		
Body weight: 40 kg	2	2.7	4
Sleep disorders	X	X	X
General adverse effects on health	-	-	X
Body weight: 60 kg	1.3	1.8	2.6
Sleep disorders	-	X	X
General adverse effects on health	-	-	-
Body Weight: 80 kg	1.14	1.3	2
Sleep disorders	-	-	X
General adverse effects on health	-	-	-

X: caffeine intake is associated with health risks (either sleep disorders or general adverse effects); -: caffeine intake is not associated with the characterized risk (neither sleep disorders nor general adverse effects).

**Table 2:** Maximum quantities of 32 mg caffeine/100 mL EDs to be consumed (ml) to prevent risks (sleep disturbances and/or general effects on health) derived from the caffeine content.

Body Weight (kg)					
40	60	80	40	60	80
Maximum quantity (ml) of EDs to be consumed to keep daily intake <1.4 mg caffeine/kg b.w. and avoid sleep disorders			Maximum quantity (ml) of EDs to be consumed to keep intake daily <3 mg caffeine/kg b.w. and avoid general adverse effects		
175 mL	262.5 mL	350 mL	375 mL	562.5 mL	750 mL

**Table 3:** EDs formulated with 2400 mg D-glucuronolactone/L: exposure assessment and risk characterization.

	Consumption Scenarios		
EDs consumption (mL)	250	333	500
D-glucuronolactone intake (mg/day)	600	800	1200
Body weight (bw)	**D-glucuronolactone** **Intake by b.w.** **(mg/kg b.w.)**		
40 kg	15.0	20	30.0
60 kg	10.0	13.3	20.0
80 kg	7.5	10.0	15.0
Body weight (bw)	**D-glucuronolactone** **Margin of Safety (MOS)**		
40 kg	66.7	50	33.3
60 kg	100	75	50
80 kg	133.3	100	66.7

**Table 4:** 4000 mg taurine/l EDs: exposure assessment (EDI).

	EDs Consumption Scenarios		
EDs volume (mL)	250	333	500
Taurine Intake (mg/day)	1000	1332	2000
Body weight (kg) and Reference intake (mg/kg b.w./day) [29]	**Taurine Estimated Dietary Intake (EDI) per b.w.** **(mg/kg b.w./day)**		
40 kg (35 mg/kg b.w./day)	25	33.3	50.0
60 kg (23.3 mg/kg b.w./day)	16.7	22.2	33.3
80 kg (17.5 mg/kg b.w./day)	12.5	16.7	25

## References

[1] Flotta D., Micò R., Nobile C.G., Pileggi C., Bianco A., Pavia M. (2014). Consumption of energy drinks, alcohol and alcohol-mixed energy drinks among Italian adolescents. Clin. Exp. Res..

[2] Nowak D., Jasionowski A. (2015). Analysis of the Consumption of Caffeinated Energy Drinks among Polish Adolescents. Int. J. Environ. Res. Public Health.

[3] Costa B.M., Hayley A., Miller P. (2016). Adolescent energy drink consumption: An Australian perspective. Appetite.

[4] Majori S., Pilati S., Gazzani D., Paiano J., Ferrari S., Sannino A., Checchin E. (2018). Energy drink and ginseng consumption by Italian university students: A cross-sectional study. J. Prev. Med. Hyg..

[5] Degirmenci N., Fossum I.N., Strand T.A., Vaktskjold A., Holten-Andersen M.N. (2018). Consumption of energy drinks among adolescents in Norway: A cross-sectional study. BMC Public Health.

[6] Galimov A., Hanewinkel R., Hansen J., Unger J.B., Sussman S., Morgenstern M. (2019). Energy drink consumption among German adolescents: Prevalence, correlates and predictors of initiation. Appetite.

[7] Vercammen K.A., Koma J.W., Bleich S.N. (2019). Trends in Energy Drink Consumption Among, U.S. Adolescents and Adults, 2003-2016. Am. J. Prev. Med..

[8] Silva-Maldonado P., Arias-Rico J., Romero-Palencia A., Román-Gutiérrez A.D., Ojeda-Ramírez D., Ramírez-Moreno E. (2022). Consumption Patterns of Energy Drinks in Adolescents and Their Effects on Behavior and Mental Health: A Systematic Review. J. Psychosoc. Nurs. Ment. Health Serv..

[9] Nadeem I.M., Shanmugaraj A., Sakha S., Horner N.S., Ayeni O.R., Khan M. (2021). Energy Drinks and Their Adverse Health Effects: A Systematic Review and Meta-analysis. Sports Health.

[10] Tomanic M., Paunovic K., Lackovic M., Djurdjevic K., Nestorovic M., Jakovljevic A., Markovic M. (2022). Energy Drinks and Sleep among Adolescents. Nutrients.

[11] Khouja C., Kneale D., Brunton G., Raine G., Stansfield C., Sowden A., Sutcliffe K., Thomas J. (2022). Consumption and effects of caffeinated energy drinks in young people: An overview of systematic reviews and secondary analysis of UK data to inform policy. BMJ Open.

[12] European Food Safety Authority (EFSA) (2015). The food classification and description system FoodEx 2 (revision 2). EFSA J..

[13] Zucconi S., Volpato C., Adinolfi F., Gandini E., Gentile E., Loi A., Fioriti L. (2013). Gathering consumption data on specific consumer groups of energy drinks. EFSA Support. Publ..

[14] Observatorio Español de las Drogas y las Adicciones (OEDA) Encuesta Sobre Uso de Drogas en Enseñanzas Secundarias en España (ESTUDES), 1994–2021. https://pnsd.sanidad.gob.es/profesionales/sistemasInformacion/sistemaInformacion/pdf/ESTUDES_2021_Informe_de_Resultados.pdf.

[15] Mattioli A.V., Farinetti A. (2021). Energy drinks and medical students: Bad drinking during COVID-19 quarantine. Clin. Nutr. ESPEN.

[16] Mattioli A.V., Sabatini S. (2021). Changes in energy drink consumption during the COVID-19 quarantine. Appl. Nurs. Res..

[17] De Sanctis V., Soliman N., Soliman A.T., Elsedfy H., Di Maio S., El Kholy M., Fiscina B. (2017). Caffeinated energy drink consumption among adolescents and potential health consequences associated with their use: A significant public health hazard. Acta Biomed..

[18] Cruz Muñoz V., Urquizu Rovira M., Valls Ibañez V., Manresa Domínguez J.M., Ruiz Blanco G., Urquizu Rovira M., Toran P. (2020). Consumo de bebidas refrescantes, deportivas y energéticas en adolescentes. Estudio BEENIS. An. Pediatr..

[19] Oliver Anglès A., Camprubí Condom L., Valero Coppin O., Oliván Abejar J. (2020). Prevalencia y factores asociados al consumo de bebidas energéticas en jóvenes de la provincia de Barcelona. Gac. Sanit..

[20] Trapp G., Allen K., O’Sullivan T.A., Robinson M., Jacoby P., Oddy W.H. (2014). Energy drink consumption is associated with anxiety in Australian young adult males. Depresss Anxiety.

[21] Trapp G., Hurworth M., Christian H., Bromberg M., Howard J., McStay C., Ambrosini G., Martin K., Harray A., Cross D. (2021). prevalence and pattern of energy drink intake among Australian adolescents. J. Hum. Nutr. Diet..

[22] Polak K., Dillon P., Koch J.R., Miller Jr W.G., Thacker L., Svikis D. (2016). Energy drink use is associated with alcohol and substance use in eighth, tenth, and twelfth graders. Prev. Med. Rep..

[23] Ruiz L.D., Scherr R.E. (2019). Risk of Energy Drink Consumption to Adolescent Health. Am. J. Lifestyle Med..

[24] Cofini V., Cecilia M.R., Di Giacomo D., Binkin N., Di Orio F. (2019). Energy drinks consumption in Italian adolescents: Preliminary data of social, psychological and behavioral features. Minerva Pediatr..

[25] Higgins J.P., Tuttle T.D., Higgins C.L. (2010). Energy beverages: Content and safety. Mayo Clin. Proc..

[26] Rubio C., Cámara M., Giner R.M., González M.J., López E., Morales F.J., Moreno M., Portillo M.P., Comité Científico AESAN (Grupo de Trabajo) (2021). del Comité Científico de la Agencia Española de Seguridad Alimentaria y Nutrición (AESAN) sobre los riesgos asociados al consumo de bebidas energéticas. Rev. Com. Científico AESAN.

[27] French Agency for Food, Environmental and Occupational Health & Safety (ANSES) Opinion of the French Agency for Food, Environmental and Occupational Health & Safety on the Assessment of Risks Concerning the Consumption of So-Called “Energy Drinks”. https://www.anses.fr/en/system/files/NUT2012sa0212EN.pdf.

[28] Norwegian Scientific Committee for Food Safety (VKM) (2019). Risk assessment of energy drinks and caffeine. VKM Rep..

[29] European Food Safety Authority (EFSA) (2009). The use of taurine and D-glucurono-γ-lactone as constituents of the so-called “energy” drinks. EFSA J..

[30] Shao A., Hathcock J.N. (2008). Risk assessment for the amino acids taurine, L-glutamine and L-arginine. Regul. Toxicol. Pharmacol..

[31] Rotstein J., Barber J., Strowbridge B., Hayward S., Huang R., Godefroy S.B. (2013). Energy Drinks: An Assessment of the Potential Health Risks in the Canadian Context. Int. Food Risk Anal. J..

[32] Pardo-Lozano R., Alvarez-García Y., Barral-Tafalla D., Farré-Albadejo M. (2007). Cafeína: Un nutriente, un fármaco o una droga de abuso. Adicciones.

[33] Soós R., Gyebrovszki Á., Tóth Á., Jeges S., Wilhelm M. (2021). Effects of Caffeine and Caffeinated Beverages in Children, Adolescents and Young Adults: Short Review. Int. J. Environ. Res. Public Health.

[34] Da Costa B.R.B., El Haddad L.P., De Martinis B.S. (2022). Caffeine content of energy drinks marketed in Brazil. Drug Test. Anal..

[35] European Food Safety Authority (EFSA) (2015). Scientific Opinion on the safety of caffeine. EFSA J..

[36] Ehlers A., Marakis G., Lampen A., Hirsch-Ernst K.I. (2019). Risk assessment of energy drinks with focus on cardiovascular parameters T and energy drink consumption in Europe. Food Chem. Toxicol..

[37] Aguiar Jr A.S., Speck A.E., Canas P.M., Cunha R.A. (2020). Neuronal adenosine A2A receptors signal ergogenic effects of caffeine. Sci. Rep..

[38] Ferré S. (2008). An update on the mechanisms of the psychostimulant effects of caffeine. J. Neurochem..

[39] Wilson C. (2018). The clinical toxicology of caffeine: A review and case study. Toxicol. Rep..

[40] Chou T.M., Benowitz N.L. (1994). Caffeine and coffee: Effects on health and cardiovascular disease. Comp. Biochem. Physiol. C. Pharmacol. Toxicol. Endocrinol..

[41] Regulation (EU) Nº 1169/2011 of the European Parliament and of the Council of 25 October 2011 on the Provision of Food Information to Consumers, Amending Regulations (EC) No 1924/2006 and (EC) Nº 1925/2006 of the European Parliament and of the Council, and repealing Commission Directive 87/250/EEC, Council Directive 90/496/EEC, Commission Directive 1999/10/EC, Directive 2000/13/EC of the European Parliament and of the Council, Commission Directives 2002/67/EC and 2008/5/EC and Commission Regulation (EC) No 608/2004. Official Journal of the European Union L 304, 22 de noviembre de 2011, 18 a 63. https://eur-lex.europa.eu/LexUriServ/LexUriServ.do?uri=OJ:L:2011:304:0018:0063:en:PDF.

[42] (2022). UNESDA Code for the Labelling and Marketing of Energy Drinks. Initially adopted by the UNESDA Board in May 2010 Updated in October 2016, November 2017 and December 2021. https://www.unesda.eu/wp-content/uploads/2022/06/UNESDA-Code-for-the-Labelling-and-Marketing-of-Energy-Drinks_January-2022.pdf.

[43] Code of Practice for the Marketing and Labelling of Energy Drinks (Adopted-BRUSSELS, December 9, 2014). https://www.energydrinkseurope.org/wp-content/uploads/2020/01/FINAL_EDE-Code-of-Practice_clean_250914.pdf.

[44] Recomendaciones Sobre el Consumo de Bebidas Energéticas. https://www.aesan.gob.es/AECOSAN/docs/documentos/noticias/2022/recomendaciones_bebidas_energeticas.pdf.

[45] Scientific Committee on Food (SCF) Opinion of the Scientific Committee on Food on Additional Information on “Energy” Drinks (Expressed on 5 March 2003). https://www.mast.is/static/files/Uploads/document/Skyrslur/opinionscientificcommitteeenergydr0503.pdf.

[46] Brosnan J.T., Brosnan M.E. (2006). The sulfur-containing amino acids: An overview. J. Nutr..

[47] Font L., Miguel M., Aragon C.M. (2001). Behavioural consequences of the hypotaurineethanol interaction. Pharmacol. Biochem. Behav..

[48] Bkaily G., Jazzar A., Normand A., Simon Y., Al-Khoury J., Jacques D. (2020). Taurine and cardiac disease: State of the art and perspectives. Can. J. Physiol. Pharmacol..

[49] Sirdah M.M., El-Agouza I.M., Abu Shahla A.N. (2002). Possible ameliorative effect of taurine in the treatment of iron-deficiency anaemia in female university students of Gaza, Palestine. Eur. J. Haematol..

[50] Brons C., Spohr C., Storgaard H., Dyerberg J., Vaag A. (2004). Effect of taurine treatment on insulin secretion and action, and on serum lipid levels in overweight men with a genetic predisposition for type II diabetes mellitus. Eur. J. Clin. Nutr..

[51] Spohr C., Brons C., Winther K., Dyerberg J., Vaag A. (2005). No effect of taurine on platelet aggregation in men with a predisposition to type 2 diabetes mellitus. Platelets.

[52] Gutiérrez-Hellín J., Varillas-Delgado D. (2021). Energy Drinks and Sports Performance, Cardiovascular Risk, and Genetic Associations; Future Prospects. Nutrients.

[53] Babu K., Zuckerman M.D., Cherkes J.K., Hack J.B. (2011). First-onset seizure after use of an Energy Drink. Pediatr. Emerg. Care.

[54] Lourenco R., Camilo M.E. (2002). Taurine: A conditionally essential amino acid in humans? An overview in health and disease. Nutr. Hosp..

[55] Schaffer S.W., Jong C.J., Ramila K.C., Azuma J. (2010). Physiological roles of taurine in heart and muscle. J. Biomed. Sci..

[56] Zhang X., Wang X., Zhang J., Pan X., Jiang J., Li Y. (2017). Effects of taurine on alterations of neurobehavior and neurodevelopment key proteins expression in infant rats by exposure to hexabromocyclododecane. Adv. Exp. Med. Biol..

[57] Chen C., Xia S., He J., Lu G., Xie Z., Han H. (2019). Roles of taurine in cognitive function of physiology, pathologies and toxication. Life Sci..

[58] Health Canada’s Proposed Approach to Managing Caffeinated Energy Drinks. https://www.canada.ca/content/dam/hc-sc/migration/hc-sc/fn-an/alt_formats/pdf/legislation/pol/energy-drinks-boissons-energisantes-eng.pdf.

[59] Brown J., Villalona Y., Weimer J., Pickering Ludwig C., Breann T.H., Massie L., Marczinski C.A., Perdan Curran C. (2020). Supplemental taurine during adolescence and early adulthood has sex-specific effects on cognition, behavior and neurotransmitter levels in C57BL/6J mice dependent on exposure window. Neurotoxicol. Teratol..

[60] Jagim A.R., Harty P.S., Barakat A.R., Erickson J.L., Carvalho V., Khurelbaatar C., Camic C.L., Kerksick C.M. (2022). Prevalence and Amounts of Common Ingredients Found in Energy Drinks and Shots. Nutrients.

[61] Lovtidende A. Bekendtgorelse om Tilsaetning af Visse Andre Stoffer End Vitaminer og Mineraler til Fodevarer. Udgivet den 13 August 2011, Nr 888, 12 August 2011. https://www.retsinformation.dk/eli/lta/2020/1697.

[62] (2012). Zweite Verordnung zur Änderung der Fruchtsaftverordnung und Anderer Lebensmittelrechtlicher Vorschriften vom 21. https://www.buzer.de/gesetz/10173/index.htm.

[63] Repetto M., Sanz P., Jurado C., López-Artíguez M., Menéndez M., de la Pena E. Glosario de Términos Toxicológicos IUPAC (Duffus y Cols. 1993). https://aetox.es/wp-content/uploads/old/glosater/glosater.aetox.pdf.

[64] Verster J.C., Koenig J. (2018). Caffeine intake and its sources: A review of national representative studies. Crit. Rev. Food Sci. Nutr..

[65] Risk Assessment of “Other Substances” —Taurine. Opinion of the Panel on Food Additives, Flavourings, Processing Aids, Materials in Contact with Food and Cosmetics of the Norwegian Scientific Committee for Food Safety. https://vkm.no/download/18.5387be10161937390293e0f/1518616535578/Risk%20assessment%20of%20other%20substances%20%E2%80%93%20Taurine.pdf.

[66] Curran C.P., Marczinski C.A. (2017). Taurine, caffeine, and energy drinks: Reviewing the risks to the adolescent brain. Birth Defects Res..

[67] Jackson D.B., Leal W.E. (2018). Energy drink consumption and the perceived risk and disapproval of drugs: Monitoring the Future, 2010-2016. Drug Alcohol Depend..

[68] Frayon S., Wattelez G., Cherrier S., Cavaloc Y., Lerrant Y., Galy O. (2019). Energy drink consumption in a pluri-ethnic population of adolescents in the Pacific. PLoS ONE.

[69] Lebacq T., Desnouck V., Dujeu M., Holmberg E., Pedroni C., Castetbon K. (2020). Determinants of energy drink consumption in adolescents: Identification of sex-specific patterns. Public Health.

